# AMMVF-DTI: A Novel Model Predicting Drug–Target Interactions Based on Attention Mechanism and Multi-View Fusion

**DOI:** 10.3390/ijms241814142

**Published:** 2023-09-15

**Authors:** Lu Wang, Yifeng Zhou, Qu Chen

**Affiliations:** School of Biological and Chemical Engineering, Zhejiang University of Science and Technology, Hangzhou 310023, China

**Keywords:** drug–target interaction, multi-head self-attention mechanism, graph attention networks, neural tensor networks, drug repositioning

## Abstract

Accurate identification of potential drug–target interactions (DTIs) is a crucial task in drug development and repositioning. Despite the remarkable progress achieved in recent years, improving the performance of DTI prediction still presents significant challenges. In this study, we propose a novel end-to-end deep learning model called AMMVF-DTI (attention mechanism and multi-view fusion), which leverages a multi-head self-attention mechanism to explore varying degrees of interaction between drugs and target proteins. More importantly, AMMVF-DTI extracts interactive features between drugs and proteins from both node-level and graph-level embeddings, enabling a more effective modeling of DTIs. This advantage is generally lacking in existing DTI prediction models. Consequently, when compared to many of the start-of-the-art methods, AMMVF-DTI demonstrated excellent performance on the human, *C. elegans*, and DrugBank baseline datasets, which can be attributed to its ability to incorporate interactive information and mine features from both local and global structures. The results from additional ablation experiments also confirmed the importance of each module in our AMMVF-DTI model. Finally, a case study is presented utilizing our model for COVID-19-related DTI prediction. We believe the AMMVF-DTI model can not only achieve reasonable accuracy in DTI prediction, but also provide insights into the understanding of potential interactions between drugs and targets.

## 1. Introduction

The selection and determination of drug targets plays a crucial role in the early drug discovery process [1]. However, conventional wet-lab experiments for molecular drug design are typically costly, labor-intensive, and time-consuming [2], and often yield low success rates in the drug development phase [3]. With the advancements in computer science, in silico prediction of drug–target interactions (DTIs) has emerged as an essential approach for drug screening and provided a new route to drug discovery [4]. It can narrow down the search scope from an astronomically large number of potential drug candidates by evaluating the interactions between existing drugs and target proteins. This computational approach can effectively complement in vitro/in vivo experiments in a variety of ways, providing insights into drug side effect prediction and drug repositioning [5,6,7,8]. In principle, many drugs are recognized for their polypharmacological nature, enabling them to interact with multiple targets associated with either single or multiple disease pathways [9]. An existing drug which was initially developed to treat a specific disease may prove beneficial in the treatment of another disease. As a consequence, the repositioning of a clinically verified drug can significantly reduce costs and expedite the drug discovery process [10,11,12,13,14,15]. Due to the extensive accessibility of public biomedical databases, the virtual screening of DTIs can assist in identifying new associations between known drugs and their unknown off targets. Successful applications of the in silico methodology can be exemplified by the reuse of astemizole [16] and betrixaban [17]. Especially during the global spread of the highly infectious coronavirus disease 2019 (COVID-19) pandemic, the application of computational predictions of DTIs in drug repositioning could offer a promising solution [17].

So far, state-of-the-art computational methods of DTI prediction can be divided into three major categories: structure-based approaches [18], ligand-based approaches [19], and chemogenomic-based approaches [20]. Structure-based approaches and ligand-based approaches can encounter limitations in cases where the 3D structures of target proteins are unknown and the numbers of known ligands for target proteins are limited, respectively. Chemogenomic-based approaches leverage the wealth of online databases containing biological information on small drugs and target proteins to facilitate predictive purposes [4,20]. In recent years, many new chemogenomic-based approaches have been developed to improve the DTI prediction accuracy. These chemogenomic-based approaches can be classified into several categories, such as network-based methods and learning-based methods. Network-based methods, independent of the 3D structures of proteins or prior knowledge of the ligands, involve constructing a DTI network combined with similarities between drugs and proteins in a matrix form to uncover new potential targets [21]. Olayan et al. proposed a model called DDR that employs a heterogeneous drug–target graph incorporating information on known DTIs, various drug–drug similarities, and various target–target similarities [22]. In this network-based method, some challenges may remain in addressing the sparsity and high dimensionality of heterogeneous DTI networks [23]. Learning-based methods, which consider the DTI prediction task as a binary classification, can be further divided into machine-learning-based [24,25,26] and deep-learning-based [27]. While machine-learning-based methods have demonstrated efficiency and robustness, they are usually outperformed by deep-learning-based methods, which are capable of learning and capturing intricate and nonlinear patterns in DTI data through their deep and hierarchical architectures without the need for handcrafted representations of drugs and targets. Conventional machine-learning based methods, on the other hand, tend to perform poorly in classification problems, particularly on large and unbalanced datasets, possibly due to the limited number of potential key features [28].

In this context, a number of deep learning frameworks have been developed over the past few years. Öztürk et al. introduced a deep-learning-based method called DeepDTA that utilizes convolutional neural networks (CNNs) to learn only the one-dimensional SMILES (simplified molecular input line entry system) representations of drugs and the one-dimensional sequence representations of target proteins to predict the DTI binding affinity [29]. Karimi et al. proposed DeepAffinity, a semi-supervised deep learning model that unifies bidirectional recurrent neutral networks (RNNs) and CNNs to exploit both unlabeled and labeled datasets consisting of compound SMILES identities and protein sequences. By utilizing abundant unlabeled data, DeepAffinity captures long-term nonlinear dependencies among residues/atoms in compounds, resulting in accurate predictions of compound–protein affinity [30]. However, in practice, amino acid sequences tend to be long, leading to a growing number of long-distance dependencies that raise computational cost and reduce model convergence. Consequently, CNN structures typically fail to model the contextual association information for protein amino acid sequences in real biological environments, while RNN structures encounter difficulties in learning the long-dependent information when dealing with long amino acid sequences. To address these issues, the attention mechanism was introduced into many models to facilitate accurate DTI prediction [31,32,33,34,35,36], allowing them to focus on the specific parts of the input data (e.g., drug molecules and protein sequences) that are most relevant for predicting the interactions. For example, Chen et al. proposed a novel framework called TransformerCPI [31], which utilized the attention mechanism for weight mapping to assess the importance of different atoms, thereby mitigating long-distance dependencies and further reducing the model’s spatial complexity. Simultaneously, the incorporation of parallel computing techniques into the attention mechanism contributed to a reduction in computational time complexity. Cheng et al. proposed a deep learning model called MHSADTI [32], which can better extract the characteristics of proteins and drugs based on the multi-head self-attention mechanism and graph attention networks (GATs). The mechanism allows deep learning models to dynamically allocate attention to the specific regions of the protein/drug input data that are most relevant to DTIs. Despite much success being achieved by the aforementioned models in predicting DTIs, there exists a certain limitation associated with these models. In the drug–protein binary system, these models often focus solely on a one-way effect, such as the impact of drugs on target proteins, while overlooking the reverse effect. However, according to the induced-fit mechanism [37], when a drug molecule binds to its target protein, both the drug and the protein undergo conformational changes. These changes, induced by the drug–protein interaction, enable the drug molecule to fit more precisely into the binding site of the target protein, resulting in the formation of a stable drug–protein complex. Consequently, the impact of drugs on proteins should be considered as equally important to the impact of proteins on drugs. Accounting for the interactions between drugs and proteins may give rise to more accurate predictions of drug–target interactions (DTIs).

In this work, we propose a novel end-to-end deep-learning-based model called AMMVF-DTIs, which incorporates the attention mechanism and multi-view fusion to enhance the accuracy of predicting DTIs. Our method employs interaction transformers (ITMs) to model the relation between graph-level embeddings and neural tensor networks (NTNs) to model the relation between node-level embeddings. Specifically, to better handle interactive information between drugs and proteins, our model adopts two strategies. First, the node embeddings of input drug/protein information are fed into two parallel ITMs to extract their interactive features. Second, the graph embeddings, obtained from the node embeddings using the attention mechanism, are fed into NTNs to find potentially important interactions between drugs and targets. Subsequently, the multi-view interaction information, extracted and processed using both strategies, is fed into the multi-layer perceptron (MLP) to achieve DTI prediction. In our model, both the node-level embeddings and the graph-level embeddings are taken into account because the interaction between drugs and proteins is influenced by many complex factors. By considering both embeddings, we avoid solely emphasizing interactions between individual atoms and can better capture the substantial conformational changes of drugs and their targets. 

This paper is organized as follows. Section 2.1 presents and discusses our results, and it compares them with those from existing models. A series of ablation experiments are also presented in Section 2.2 to demonstrate the importance of each module in our model. Additionally, we include a case study concerning COVID-19 treatment in Section 2.3, and we examine the possible limitations of our model in Section 2.4. Section 3.1 provides an overview of the three datasets used, while Section 3.2 details all the methods employed in this work. Our findings are summarized in Section 4.

## 2. Results and Discussion

### 2.1. Performance on Human, C. elegans, and DrugBank Baseline Datasets

In this work, we used the PyTorch framework for our proposed model training on an NVIDIA RTX 3090 GPU, and we selected area under curve (AUC), recall, and precision as the three main metrics to evaluate the model’s performance. During the network training process, we employed Xavier weight initialization [38] to avoid the issue of vanishing gradients and used Adam [39] for optimizing the loss function to address the problem of gradient oscillation. The optimal hyperparameters for the model are presented in Table 1.

To validate the effectiveness of our proposed model AMMVF-DTI, we performed our model on two datasets (human and *C. elegans*), and we selected ten popular models for comparison on the human dataset and nine popular models for comparison on the *C. elegans* dataset. These models fall into two categories: machine-learning-based methods including K-nearest neighbor (KNN), RF, L2 logistic (L2), SVM, and deep-learning-based methods including MDL-CPI [40], graph neural network (GNN) [41], graph convolutional network (GCN), GraphDTA [42], DrugVQA (VQA-seq) [43], and TransformerCPI [31]. The results are shown in Table 2 and Table 3. Moreover, to validate the generalization of AMMVF-DTI, we also performed our model on the DrugBank dataset and conducted comparative studies with five existing models including RWR [44], DrugE-Rank [45], DeepCPI [46], DeepConv-DTI [47], and MHSADTI [32]. A five-fold cross-validation study was conducted, and the average values were used as the final results for AUC, precision, and recall, as shown in Table 4. Many reference data for comparison were directly taken from the literature [31,40,48], and the best performance values are highlighted in bold.

Table 2 shows that our AMMVF-DTI model outperformed all competing models in terms of AUC and precision on the human dataset. Although the recall of our model was slightly lower than that of SVM or DrugVQA, it still outperformed that of the other eight models. Specifically, AMMVF-DTI achieved an AUC of 0.986, precision of 0.976, and recall of 0.938, an improvement in performance over the ten competing models by 1.3–12.6%, 1.0–11.4%, and 1.0–14.0%, respectively. These substantial improvements clearly indicated the effectiveness of our model. While SVM achieved the best recall value on the relatively small human dataset, its performance in terms of recall exhibited a drastic drop on the relatively large *C. elegans* dataset, as shown in Table 3. This phenomenon may be ascribed to the fact that machine-learning-based methods rely on prior knowledge and traditional feature engineering, thereby limiting their generalization and robustness compared to deep-learning-based methods. Considering the reliability of AUC and the trade-off between precision and recall, we believe that our model exhibits the best performance in general in terms of AUC, precision, and recall.

As shown in Table 3, AMMVF-DTI achieved the best performance among all competing models on the *C. elegans* dataset, with AUC, precision, and recall reaching 0.990, 0.962, and 0.960, respectively. These values represented improvements in AUC, precision, and recall by 0.2–13.2%, 1.0–16.1%, and 0.7–14.2%, respectively, over the other nine competing models. This excellent performance shows that our model has a powerful ability to distinguish positive samples from negative ones, low classification error rates, and high robustness. First, the AUC value close to 1 indicates that the classifier can accurately rank positive samples above negative samples. With an AUC of 0.990, our classifier almost perfectly separates positive and negative samples. Second, as the AUC value approaches 1, the classification error rate becomes lower, indicating that the classifier hardly ever misclassifies negative samples as positive ones or vice versa. Third, the AUC value, which provides an overall evaluation of classifier performance, is not affected by the choice of classification thresholds. Therefore, an AUC of 0.990 shows the outstanding performance and high robustness of our model across many classification thresholds. Furthermore, our AMMVF-DTI model demonstrates improved and well-balanced precision and recall, which indicates that the model possesses a stable ability to identify different categories of data and delivers more reliable classification results.

Combining Table 2 with Table 3, we can observe a trend; as the dataset size increases, the performance of machine learning models declines, while deep learning models show significant overall improvement. This can be attributed to deep learning’s utilization of end-to-end learning, which replaces feature engineering with automatic extraction of more complex and abstract semantic expressions. As a result, deep learning models become more automated and more adaptable to complex data distributions, enhancing their fitting results. Our AMMVF-DTI model exhibited better performance on the *C. elegans* dataset than on the human dataset. This outcome serves as strong evidence for the model’s reliability and capacity for generalization. By effectively adapting to the characteristics of the *C. elegans* dataset, our model demonstrated its capacity to handle diverse data types and further validated its robustness.

As is shown in Table 4, our AMMVF-DTI model exhibited significant improvements over five competing models in all the performance metrics on the larger and more diverse DrugBank dataset. Specifically, our model improved AUC by 9.42–19.75%, precision by 11.43–19.88%, and recall by 11.66–25.73%, over the five competing models. It is suggested that our model exhibited good classification performance, which further demonstrates its robustness and capacity for generalization.

The above analysis demonstrates that our model can effectively capture independent and complex interactive features, leading to more accurate DTI prediction. We owe the excellent performance of AMMVF-DTI to two key factors. First, by combining GAT with the multi-head self-attention mechanism, we successfully address the challenge of extracting context-related information. Different weight values are assigned to neighboring nodes, avoiding the impact of noise data connections on important nodes and hence improving the overall model performance. Second, AMMVF-DTI utilizes both node-level and graph-level embeddings, allowing for consideration of detailed features and attention to the global structure. The model successfully mines *K* potential interaction features in different spaces, preventing the loss of local features and important interaction characteristics. This enhances the model’s feature fitting and contributes to its significant performance improvement.

### 2.2. Ablation Study

**Figure 1:**
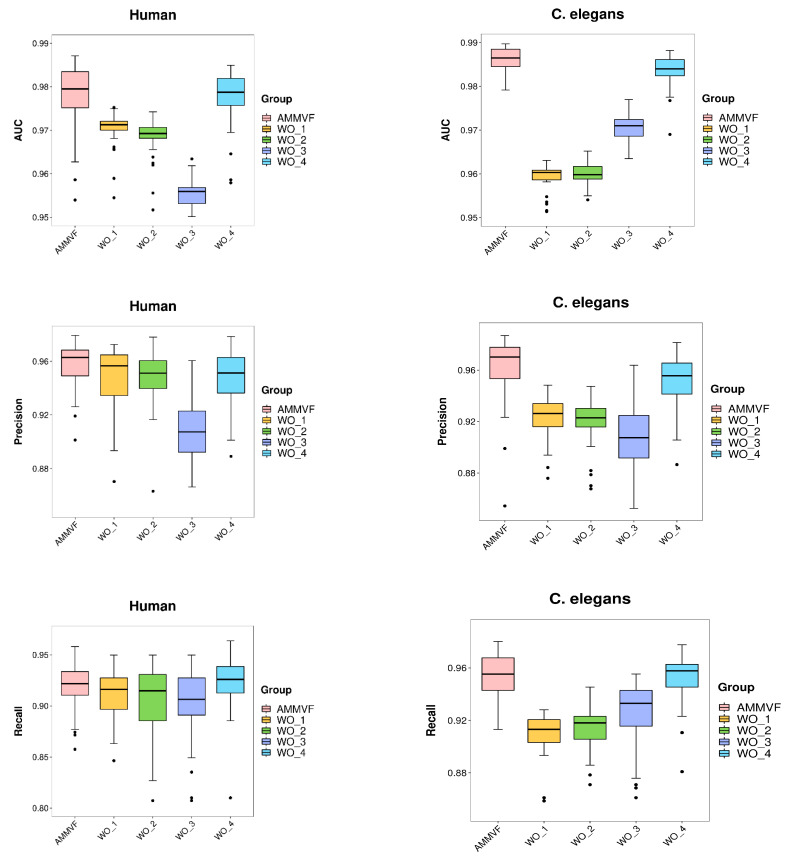
Results of ablation experiments on the human and *C. elegans* datasets in terms of AUC, precision, and recall.

To validate the rationale and necessity of each module in our model, the following ablation experiments were conducted for five different models: (1) the proposed model AMMVF-DTI with all modules intact; (2) AMMVF-DTI without interactive networks NTN and ITM, only using independent features; (3) AMMVF-DTI without ITM, only using graph-level interaction features; (4) AMMVF-DTI without NTN, only using node-level interaction features; and (5) AMMVF-DTI without ATT, where the graph-level embedding was obtained by using global average operation directly. In Figure 1, AMMVF represents AMMVF-DTI (intact), WO_1 represents AMMVF-DTI (without NTN and ITM), WO_2 represents AMMVF-DTI (without ITM), WO_3 represents AMMVF-DTI (without NTN), and WO_4 represents AMMVF-DTI (without ATT).

Figure 1 shows the results of the ablation experiments on the human and *C. elegans* datasets. Overall, our proposed model AMMVF-DTI (intact) exhibited much better performance than AMMVF-DTI (without NTN and ITM) on both datasets, which proves that directly concatenating independent features makes it difficult for the model to learn complex drug–target interactions, leading to important information loss. Therefore, the interactive networks ITM and NTN play a crucial role in improving performance. It was also found that both AMMVF-DTI (without ITM) and AMMVF-DTI (without NTN) perform in general much worse than AMMVF-DTI (intact). This confirms the importance of both graph-level and node-level interactions. Removing the node-level interaction module results in the loss of important details, such as the interactions between atoms, while removing the graph-level interaction module leads to the loss of the global structure. In our work, node-level embeddings represent individual residues or atoms, while protein pockets (binding sites) are a domain whose conformation is likely to be affected by surrounding amino acid residues, so that conformational adjustment would occur during ligand binding. Consequently, dynamically capturing global features significantly impacts the model’s robustness. In addition, AMMVF-DTI (intact) achieved better results than AMMVF-DTI (without ATT) because the attention module can better explore the important substructures of drug and protein sequences. The multi-head mechanism captures multiple spatial and functional relationships, which might be consistent with real chemical and biological phenomena, such as hydrophobic interactions, interactions between non-covalent atoms, and hydrogen bonding between amino acids.

It is still worth noting that, on the human dataset, the recall value showed no significant difference between the five models tested. This suggests that a relatively robust feature representation was learned from each module on the smaller dataset. Even with the absence of any specific module, the remaining modules can still effectively extract relevant and useful information, resulting in satisfactory performance and indicating the strong adaptability of each module. However, on the larger *C. elegans* dataset, the results showed significant differences, underscoring the necessity and rationality of each module. The performance variation on the human and *C. elegans* datasets indicates that each module plays a crucial role in handling more complex and diverse data, which confirms the importance of their integration within our complete model AMMVF-DTI.

### 2.3. Case Study

**Figure 2:**
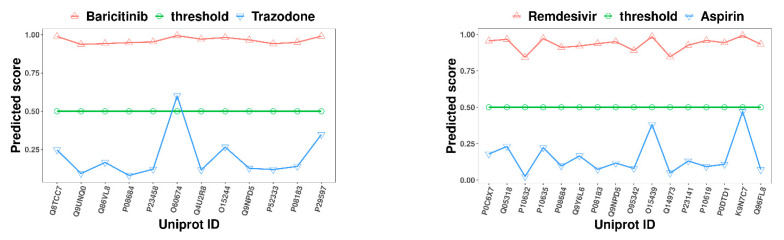
Predicted scores of COVID-19-related DTIs using our AMMVF-DTI model. On the **left**, red triangles represent the interactions between the drug baricitinib and its 12 target proteins, while blue inverted triangles represent the interactions between the unrelated drug trazodone and the same proteins. On the **right**, red triangles represent the interactions between the drug remdesivir and its 16 target proteins, while blue inverted triangles represent the interactions between the unrelated drug aspirin and the same target proteins.

To validate the effectiveness of the AMMVF-DTI model in drug repurposing [49], we employed our model AMMVF-DTI to predict COVID-19-related DTIs. Relevant interaction information concerning COVID-19-related drugs were screened from the DrugBank database [50]. Two inhibitors, baricitinib [51,52] and remdesivir [53], were selected, along with two unrelated drugs, trazodone and aspirin. Searching from the DrugBank database, we obtained a total of 12 target proteins for baricitinib and a total of 16 target proteins for remdesivir. These proteins included members from the SLC family, cytochrome P450 enzyme family, JAK family, ABC transporter family, solute carrier organic anion transporter family, fatty acid esterase family, replicase polyprotein 1ab, RNA polymerase L, and lysosome protective protein. For constructing the dataset in this case study, we obtained the SMILES sequences of drug molecules and amino acid sequences of targets from the DrugBank database, and then fed these data into the AMMVF-DTI model to predict interactions. The predicted scores of the interactions between the two inhibitors and their respective protein targets are shown in Figure 2.

The results in Figure 2 show that the scores of the interactions between the two inhibitors and their respective protein targets ranged from 0.91 to 0.99. With a threshold set at 0.5, a value indicating high confidence, all the DTI predictions were correct, further confirming the effectiveness of our model. As for the two unrelated drugs and targets, their score values were mainly distributed in the range of 0.02 to 0.47, with only one prediction score of 0.59 slightly above the threshold. However, this value was still within an acceptable range, displaying no significant deviation. These predictions shed light on how drugs interact with targets associated with COVID-19, aiding in the understanding of DTIs and offering guidance for further drug design optimization.

Accordingly, implementing the task of drug repurposing using our model AMMVF-DTI may involve the following steps. First, the model should be trained on large public datasets (e.g., DrugBank) to learn general patterns and relationships in DTIs, making it more likely that it will perform well on a wide range of drugs and targets. Second, known DTI data pertinent to a new medical condition should be screened and acquired to construct a relevant dataset, and these can be derived from public databases or sources in the literature. Lastly, based on the model’s prediction scores, we can identify potential drug candidates with potential therapeutic efficacy. However, it should be noted that deep learning models can serve as valuable auxiliary tools but should not be considered the sole decision-making basis. In ensuring the effectiveness of drugs, biological experimental validation of the selected drug candidates must be conducted.

### 2.4. Limitations

Although our model achieved reasonable accuracy in DTI prediction, there are certain limitations associated with the method. First, as shown in Table 1, we utilized a three-layer GAT to capture features of atoms and their two adjacent atoms within the graph, including information about the atoms and their interactions. This may result in the loss of information related to the cyclic structures of certain drug molecules, and therefore adding more layers would help the GAT module learn increasingly abstract representations of the entire graph [54]. Secondly, our model utilized 1D SMILES representations for drug molecules and 1D sequences for target proteins, leading to a general neglect of atomic interactions within both the drug molecules and target proteins. Given the structural complexity inherent in drug molecules and target proteins, the introduction of 3D interaction graphs is a necessity for achieving more precise DTI predictions [55]. Finally, to extract more comprehensive and effective features, we adopted a multi-vision fusion approach, which, to some extent, increased the computational cost. Therefore, determining the optimal trade-off between computational cost and prediction accuracy should be task-specific and requires further investigation.

## 3. Materials and Methods 

### 3.1. Datasets

In most supervised DTI prediction tasks, datasets typically comprise experimentally validated positive samples (representing interactions between drugs and targets) and randomly selected negative samples (representing non-interactions between drugs and targets). However, the randomly generated negative samples may contain unknown positive samples, which can lead to overfitting of the model [56]. Hence, the selection of appropriate and effective negative samples is crucial in constructing datasets. In this study, we sought to evaluate our proposed model using two benchmark datasets (human and *C. elegans*) generated by Liu et al. [28]. The positive samples in both datasets were constructed from the manually curated databases DrugBank [50] and Matador [57], while negative samples were created by an effective screening method based on dissimilar rules [28]. To further validate the accuracy and generation of our model, we also performed our work on a larger dataset, DrugBank. The positive-to-negative sample ratios for all three datasets were approximately 1:1. A summary of the balanced datasets used in our work is shown Table 5.

All three datasets utilize SMILES strings to represent drugs and amino acid sequences to represent proteins. For drugs, the maximum SMILES string lengths in the human, *C. elegans*, and DrugBank datasets are approximately 420, 252, and 250 characters, respectively. On average, the SMILES string lengths in the human, *C. elegans*, and DrugBank datasets are about 47, 34, and 55 characters, respectively. For target proteins, the maximum sequence lengths in the human, *C. elegans*, and DrugBank datasets are 5038, 13,100, and 14,507 characters, respectively, with average sequence lengths of around 623, 530, and 545 characters, respectively.

**Figure 3:**
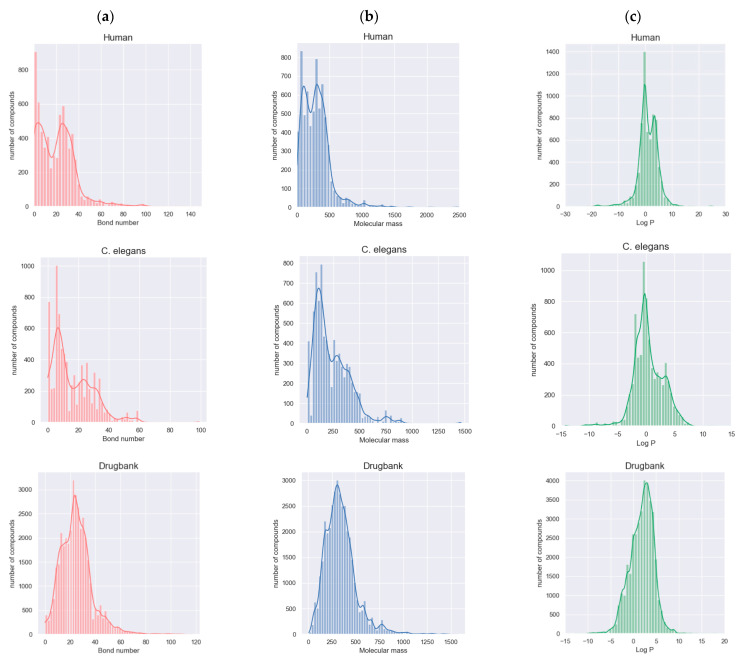
Distribution of (**a**) the bond numbers of drug molecules in the three datasets (**left** panel), (**b**) the molecular masses of drug molecules in the three datasets (**middle** panel), and (**c**) the partition coefficients of drug molecules (LogP) in the three datasets (**right** panel).

In a DTI prediction task, several molecular descriptors are also essential for accurately assessing whether a drug molecule will interact with a specific target protein, as well as the strength of that interaction, such as bond number, molecular mass, and partition coefficient. The bond number can reflect the structural diversity of drug molecules in a specific dataset, while the mass of a drug molecule can affect its pharmacokinetics and bioavailability. The partition coefficient of a drug molecule between aqueous and lipophilic phases, most commonly referred to as the LogP, can represent the lipophilicity of the molecule, impacting its solubility and ability to cross cell membranes. The distributions of the bond numbers, molecular masses, and partition coefficients of the drug molecules in the three datasets are shown in Figure 3.

### 3.2. Methods

In DTI prediction, the interaction between drugs and targets is inherently complex. Simply concatenating the independent characteristics of molecules for downstream tasks can lead to challenges in effectively learning essential information, thereby impacting its generalization and performance accuracy. Additionally, in our study, drug atoms and protein residues were represented at node-level, but focusing solely on interactions from this node-level perspective might result in a loss of integrity, potentially magnifying minor effects in black box learning. Inspired by a recent work of Bai et al. [58], we introduced two strategies to consider drug–target interactions: one was to adopt the ITM module based on interactive feature extraction of node-level embeddings, and the other was to use the NTN module based on relationship mining between graph-level embeddings.

**Figure 4:**
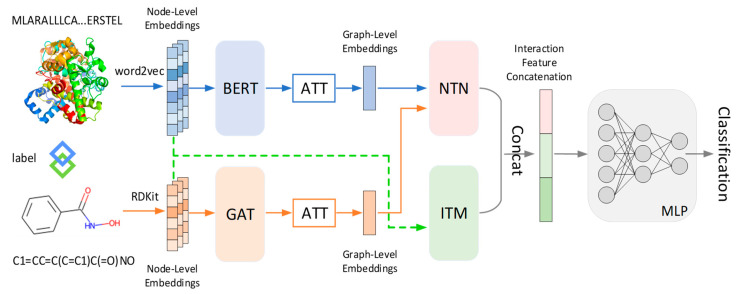
Framework of the proposed model AMMVF-DTI, which consists of three core modules: (1) feature extraction modules (BERT/GAT/ATT), (2) interaction information extraction modules (ITM/NTN), and (3) prediction module (MLP).

Figure 4 shows the framework of our proposed AMMVF-DTI model, consisting of three core modules: (1) the feature extraction modules, which included bidirectional encoder representations from the transformers (BERT), GAT, and attention (ATT) modules; (2) the interaction information extraction modules, which included ITM and NTN modules; and (3) the prediction module, which consisted of an MLP module. A detailed description of the flowchart can be outlined as follows. First, the model takes drug SMILES strings and protein sequences as input and employs the chemical information toolkit RDKit and the pre-trained Word2Vec model [59] to obtain node-level representations of the drug and protein molecules, respectively. Next, the GAT and BERT modules are utilized to extract features from the drug and protein molecules, respectively. These features are then fed into the ATT module, enabling the generation of graph-level representations of the molecules. The node-level and graph-level representations of the molecules are then processed by the ITM and NTN modules, respectively, which can effectively capture the information about interactions between the drugs and proteins. Finally, the DTI classification prediction is performed using the MLP module. The Python code for our model AMMVF-DTI is included in the Supplementary Materials.

#### 3.2.1. BERT Module

**Figure 5:**
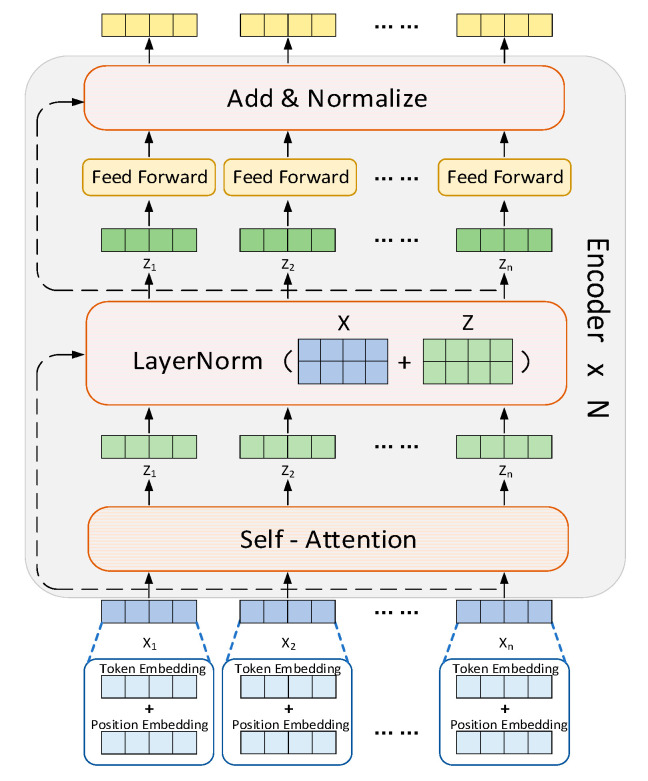
Structure of the BERT module, which consists of three parts: input embedding with multiple transformer encoding layers, a multi-head self-attention mechanism, and a feedforward neural network. First, the input vector X_n_ is transformed into vector Z_n_ through multi-head self-attention, and the two vectors are then added together using a residual connection. Subsequently, layer normalization and linear transformation are applied to the vectors to enhance the model’s capacity to capture complex patterns.

BERT is a natural language processing (NLP) model that was introduced by Google [60]. Designed to handle sequential data efficiently, BERT was built on the transformer architecture [61], which relies on a self-attention mechanism to capture contextual relationships between words in a sentence. The robustness of the BERT model in predicting DTIs has already been proved [62]. In this work, the BERT framework was considered as a feature extractor for target proteins, not a pre-training model. Figure 5 shows the structure of a BERT network, which consists of three parts: input embedding, a multi-head self-attention mechanism, and a feedforward neural network.

Firstly, BERT employs token embeddings and positional embeddings to convert each text unit (token) into a one-hot vector representation while preserving semantic and positional information. Token embedding encodes each token into a fixed-dimensional semantic representation, allowing the model to capture the meaning of individual tokens. However, since the transformer architecture lacks a natural sense of position due to its non-recursiveness, positional embedding is introduced to model the temporal characteristics of each token’s position in the input sequence. This ensures that BERT can better understand the contextual information and relationships among the tokens within the sequence. The positional encoding is given as follows:

(1) $$p(k,2i)=\sin(\frac{k}{n^{2i/d}})$$

(2) $$p(k,2i+1)=\cos(\frac{k}{n^{2i/d}})$$

In Equations (1) and (2), *p* represents a position function based on the periodic characteristics of sine and cosine functions, *k* the position index in the input sequence, *i* the index of the position dimension, *d* the dimensionality of the output position coding vector, and *n* a user-defined quantity set to be 10,000. Therefore, when the offset *m* is introduced in the positional embeddings, the positional encoding can be obtained as follows:

(3) $$p(k+m,2i)=\sin(\frac{k}{n^{2i/d}})\times \cos(\frac{m}{n^{(2i+1)/d}})+\cos(\frac{k}{n^{(2i+1)/d}})\times \sin(\frac{m}{n^{2i/d}})$$

(4) $$p(k+m,2i)=-\sin(\frac{k}{n^{2i/d}})\times \sin(\frac{m}{n^{2i/d}})+\cos(\frac{k}{n^{(2i+1)/d}})\times \cos(\frac{m}{n^{(2i+1)/d}})$$

As shown in Equations (3) and (4), the positional feature vector is a linear operation between the vectors of positions *k* and *m*, indicating that this position contains relative positional information. As a result, the attention mechanism can calculate weight values (the dot product of the two vectors) without being influenced by absolute positions. The multi-head self-attention mechanism then leverages the contextual information to capture more comprehensive feature representations. Finally, the feedforward neural network utilizes an activation function *Relu* and two learnable parameter matrices (*W*_1_∈ℝ*^d^*^hid×*d*^ and *W*_2_∈ℝ*^d^*^×*d*hid^, where *d*_hid_ represents the dimension of feature vectors in the hidden layers) to obtain the ultimate high-level semantic feature vector of the BERT model using Equation (5):

(5) $$MLP(x)=W_{2}(relu(W_{1}x+b_{1}))+b_{2}$$

#### 3.2.2. GAT Module

Although graph-based studies have been favored by researchers in recent years, it is still challenging to represent the characteristics of graph structures [63,64,65]. GAT, a type of neural network architecture designed for processing data in the form of graphs, introduces an attention mechanism to allow each node to selectively attend to its neighbors during the learning process. First, the atom list (including aromaticity) and the adjacency matrix are constructed, represented by the original input of the mapping data. The length of the atom list corresponds to the total number of atoms in the drug molecule *n*_atom_, and the size of the adjacency matrix *W*_adj_ is equal to *n*_atom_ × *n*_atom_. Through the embedding layer, a node feature matrix with a size of *n*_atom_ × *dim* is obtained, where *dim* represents the atomic embedding dimension. Finally, during feature concatenation, a self-attention mechanism is applied to dynamically weigh the importance of neighboring atoms and the attention coefficient *e_ij_*, which indicates the importance of atom *i* to atom *j* in the graph, can be obtained as follows:

(6) $$e_{ij}=a(Wh_{i},Wh_{j}),j\in N_{i}$$

where *h_i_* and *h_j_* denote atom *i* and atom *j*, respectively; *W* represents a trainable weight matrix, which is used for dimension enhancement mapping of atomic features; *N_i_* is the neighborhood of atom *i* in the graph; and *a* indicates the correlation coefficient. The weight value can be obtained by mapping the learnable matrix *α*^T^ with the size of (2·*dim*) × 1. The *LeakyRelu* activation function [66] is used to calculate the attention coefficient:

(7) $$e_{ij}=LeakyRelu(\alpha^{T}[Wh_{i}||Wh_{j}])$$

where || represents the concatenated operation. Then, we use the *softmax* function to normalize the attention coefficient:

(8) $$a_{ij}=softmax(e_{ij})=\frac{\exp(e_{ij})}{\Sigma_{k\in N_{i}}\exp(e_{ik})}$$

Combining Equations (6)–(8), we can obtain the correlation coefficient using

(9) $$a_{ij}=\frac{\exp(LeakyRelu(\alpha^{T}[Wh_{i}||Wh_{j}]))}{\sum_{k\in N_{i}}\exp(LeakyRelu(\alpha^{T}[Wh_{i}||Wh_{k}]))}$$

Subsequently, the normalized attention coefficient is used to update the hidden atomic vector *h_i_*, resulting in *h_i_*^′^,

(10) $$h_{i}^{\prime }=\sigma (\sum_{j\in N_{i}}\alpha_{ij}Wh_{j})$$

where σ is the *Relu* activation function. Finally, in order to make the self-attention learning process more stable, we extend the multi-head mechanism to let each head learn features in different spaces. After *K* independent attention mechanisms are performed, *h_i_*^′^(*K*) is obtained by feature concatenation:

(11) $$h_{i}^{\prime }(K)=\overset{K}{\underset{k=1}{\left|\right|}}\sigma (\sum_{j\in N_{i}}\alpha_{ij}Wh_{j})$$

#### 3.2.3. ATT Module

After processing proteins and drugs through the BERT and GAT networks, respectively, advanced semantic features are obtained. These features serve as input to the ATT module, which aims to decrease reliance on external information, more effectively capture internal correlations, and obtain graph-level representations of molecules. This module consists of two multi-headed self-attention layers and two fully connected layers. Initially, the input features are embedded through an activation function, converting them into a tensor of (batch_size, length, hid_dim), where batch_size represents the batch input size, length represents the number of atoms (or sequence length), and hid_dim represents the hidden layer feature dimension. These two tensors are then fed to multiple self-attention layers for self-attention calculation and an average pooling operation, which allows the model to focus on relevant information and aggregate important features for each atom (or sequence element). Finally, the hyperbolic tangent function is applied to obtain the global vector representations for drugs and proteins and return them as the output of the ATT module. A learnable weight matrix is also used in the model to transform the global vector representation into lower-dimensional vectors to reduce the complexity of the model. 

#### 3.2.4. ITM Module

**Figure 6:**
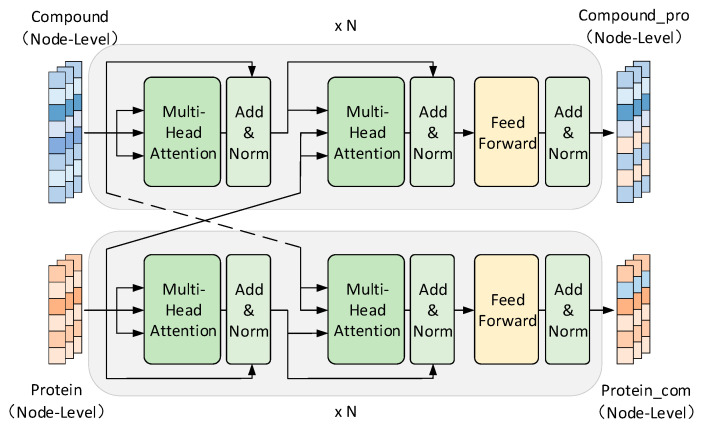
An overview of the structure of the ITM module. “Add” represents residue connection, “Norm” represents normalization, and “× N” represents the number of layers in the cross-attention mechanism.

Inspired by the works of Li et al. [67] and Chen et al. [68], we developed an ITM module that consists of two parallel interacting transformer encoders. This module serves to extract node-level interaction features between drugs and targets. Figure 6 shows the structure of the module, which includes the multi-head cross-attention mechanism, the residual connection, and layer normalization.

We first employ the pre-trained Word2Vec model to input *X_embedding_* as the node-level features for ITM, and then construct query, key, and value matrices through self-attention operations, denoted as *Q* = {*q*_1_^T^, *q*_2_^T^, …, *q_l_*^T^}, *K* = {*k*_1_^T^, *k*_2_^T^, …, *k_l_*^T^}, and *V* = {*v*_1_^T^, *v*_2_^T^, …, *v_l_*^T^}, where *l* represents the sequence length, *q*_i_^T^∈ℝ^dq^, *k*_i_^T^∈ℝ^dk^, and *v*_i_^T^∈ℝ^dv^. In order to improve model fitting, a trainable weight matrix *W* is used for linear mapping of the input by the following equation:

(12) $$\begin{array}{l} Q=X_{embedding}\times W_{Q}\\ K=X_{embedding}\times W_{K}\\ V=X_{embedding}\times W_{V} \end{array}$$

In the cross-attention layers of ITM, the sequence information of another encoder is embedded in the multi-head scaled dot attention block of the decoder. Specifically, two transformers are used to process drug (or compound) and protein input information independently. In the interaction layer, the drug embedding sequence (*E_drug_*) serves as the query matrix (*Q_drug_*) for the multi-head scaled dot attention block, while the protein embedding sequence (*E_pro_*) serves as the key matrix (*K_pro_*) and value matrix (*V_pro_*) input to the interaction layer. The correlation score in the interaction layer is determined based on the vector dot product value. A larger dot product value indicates a higher similarity between the drug and protein embeddings, suggesting a stronger correlation between them. Therefore, the correlation score is calculated using:

(13) $$Attention(Q_{drug},K_{pro},V_{pro})=softmax(\frac{Q_{drug}K_{pro}}{\sqrt{d}})V_{pro}$$

where *d*^1/2^ represents the scaling factor, which is used to adjust the scaling of the dot product attention mechanism [38], so that the “steepness” of the *softmax* distribution is decoupled from *d*, thereby avoiding a vanishing or exploding gradient during the training process. By appropriately scaling the dot product, the attention mechanism becomes more stable and facilitates effective learning. In order to enhance the robustness and efficiency of this module, a multi-head mechanism is used to map the input to different subspaces, learn multiple semantic features, concatenate the results, and perform linear transformations. The calculation for the multi-head mechanism is as follows:

(14) $$MultiHead(Q_{1\ldots n},K_{1\ldots n},V_{1\ldots n})=Concat(head_{1},head_{2}\ldots head_{n})W^{O}$$

where *head_i_* = *Attention* (*QW_i_^Q^*, *KW_i_^K^*, *VW_i_^V^*), *W^O^*∈ℝ^(*n*·*d*^*^v^*^)×*d*^ is a learnable matrix used for feature mapping, and *n* represents the number of multiple heads. The outputs from all heads are concatenated using the *Concat* operation to combine individual self-attention mechanism vectors, resulting in the final DTI feature vector.

In addition to the multi-head cross-attention mechanism, residual connection and layer normalization are incorporated to enhance module stability and improve fitting at the input and output ends, respectively. The residual connection plays a crucial role when the learning ability reaches saturation. It breaks the symmetry of the network and introduces identity mapping, enabling data to flow across layers: *f*_n+1_ (*x*) = *x* + *f*_n_ (*x*). To further enhance training efficiency, we apply layer normalization to the activation values of the hidden layers. This normalization operation helps maintain a standard normal distribution of the data, accelerating the training process and facilitating quicker convergence during model training.

#### 3.2.5. NTN Module

**Figure 7:**
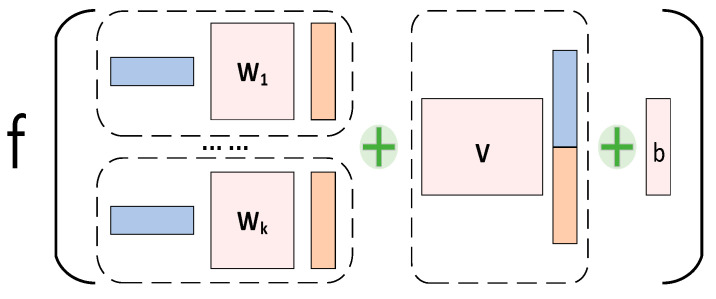
An overview of the structure of the NTN module.

Since effective graph-level embedding can capture global structural patterns, we employ the NTN strategy to explore potential *K* major associations between drugs and targets as interactive eigenvector outputs. The structure of the model is shown in Figure 7 and consists of two main parts: (1) the exploration of key factors of intermolecular *K*-dimension; (2) the fusion of molecular characteristics.

There are various molecular interactions between drugs and target proteins, such as van der Waals interactions, electrostatic interactions, cation–aromatic system interactions, and halogen bond interactions. If the module is allowed to learn all possible relationships, a large correlation table needs to be maintained. Besides the large computational cost, what the model learns may not be the essential drug–target interactions, ultimately affecting the accuracy of the model’s predictions. To address this issue, we adopted a matrix decomposition approach, which maintains two smaller matrices representing the characteristic information of the drugs and proteins. Implicit vectors (i.e., the weight parameter *W*) are utilized as the connection between the small matrices, enabling the extraction of the most valuable *K*-dimensional features. The global feature vector obtained from the ATT module serves as the input to the NTN module. A bilinear model is then used to effectively model the relationship between the drugs and proteins:

(15) $$g(h_{drug},h_{pro})=f(h_{drug}^{T}W_{\left[1:k\right]}h_{pro}+V[h_{drug}||h_{pro}]+b)$$

where *W*_[1:*k*]_∈ℝ*^d×d×k^* and *V*∈ℝ*^k×2d^* are weight matrices that can be learned as model parameters, || indicates the concatenation of two vectors to form a multimodal transport, *b*∈ℝ*^k^* is a biasing factor for tuning the model parameters, the adjustable hyperparameter *k* determines the dimension of the interaction information, and the *Relu* activation function *f* is used to obtain the interactive feature vectors.

#### 3.2.6. MLP Module

In our work, DTI prediction was characterized as a supervised binary issue. In order to make full use of the interaction information, we concatenated the characteristic outputs of the node and graph interaction network (*v_scores_*, *v_com_p_*, *v_pro_c_*) to generate the input for the downstream task prediction module. The integration of drug and protein structural information is already accomplished by the NTN module, so there is no need to further concatenate the feature vectors for the drugs and proteins again. This input is then fed into a multi-layer perceptron without bias, and the predicted value $\hat{y}$ is obtained. To train the model, we used the cross-entropy (CE) function to calculate the loss value between the true label *y* and the predicted value $\hat{y}$. The model parameters were updated through backpropagation, optimizing the performance on the DTI prediction task. The relevant calculations are as follows:

(16) $$\hat{y}=sigmoid(W_{2}relu(W_{1}concat(v_{scores,}v_{com\_p,}v_{pro\_c})))$$

(17) $$CE(y,\hat{y})=-[y\log\hat{y}+(1-y)\log(1-\hat{y})]$$

## 4. Conclusions

Accurate prediction of DTIs plays a crucial role in drug discovery and repositioning. Despite significant advancements in deep learning technology for drug research, the current predictive performance is not yet entirely satisfactory. The primary challenges faced by existing methods involve (1) better representing the independent features of drugs and their target proteins, and (2) capturing the interactions between drugs and targets more comprehensively. Addressing these challenges is essential to enhance the accuracy and reliability of DTI prediction and thereby promote drug research and development.

In this study, we proposed an end-to-end deep learning model called AMMVF-DTI that is based on an attention mechanism and multi-view interaction for DTI prediction. First, the BERT and GAT modules were used to capture the independent features of drugs and proteins. Subsequently, we employed attention scores to assess the importance of different sub-sequences of drugs and proteins in the ATT module, where the node-level features were aggregated into graph-level representations. Two interactive feature extraction modules, ITM and NTN, were then introduced at node level and graph level, respectively, to model the complex associations between drug and protein targets more effectively. Finally, all features were fused into the MLP module of the downstream task prediction of DTIs. Multiple experiments on the human, *C. elegans*, and DrugBank benchmark datasets showed that our proposed AMMVF-DTI model has better performance than many existing methods, and proved the powerful predictive ability of our model. In addition, ablation experiments were conducted on the human and *C. elegans* datasets, which confirmed the importance of each module in AMMVF-DTI. Lastly, COVID-19-related DTI prediction was selected as a case study to further demonstrate the capabilities of AMMVF-DTI in practical applications.

Although there remain some limitations in using this model, these are expected to be addressed in our future work. In practical applications, with the help of large public databases, this deep learning model can be used to analyze the proteomic data of a virus and predict potential interactions with existing drugs. This can accelerate the identification of drugs that may inhibit a disease’s effects. Also, since the performance of deep learning models heavily relies on the availability of known DTIs and data quality, it is essential to consider complementary approaches such as structure-based and ligand-based methods to address more intricate DTI challenges.

## Figures and Tables

**Table 1:** The settings of hyperparameters for our proposed model AMMVF-DTI.

Hyperparameter	Value
Epoch	40
Dropout	0.1
Learning rate	1 × 10^−3^
Regularization coefficient	1 × 10^−4^
The radius	2
The *n*-gram	3
The number of major potential associations *K*	16
The dimensions of the hidden layer	64
The number of GAT layers	3
The number of multi-head self-attention	8

**Table 2:** The performance comparison of our model AMMVF-DTI with existing models on the human dataset. The best performance values are highlighted in bold.

Model	AUC	Precision	Recall
KNN	0.860	0.927	0.798
RF	0.940	0.897	0.861
L2	0.911	0.913	0.867
SVM	0.910	0.966	**0.969**
MDL-CPI	0.959	0.924	0.905
GNN	0.970	0.918	0.923
GCN	0.956 ± 0.004	0.862 ± 0.006	0.928 ± 0.010
GraphDTA	0.960 ± 0.005	0.882 ± 0.040	0.912 ± 0.040
DrugVQA (VQA-seq)	0.964 ± 0.005	0.897 ± 0.004	0.948 ± 0.003
TransformerCPI	0.973 ± 0.002	0.916 ± 0.006	0.925 ± 0.006
AMMVF-DTI (this work)	**0.986**	**0.976**	0.938

**Table 3:** The performance comparison of our model AMMVF-DTI with existing models on the *C. elegans* dataset. The best performance values are highlighted in bold.

Model	AUC	Precision	Recall
KNN	0.858	0.801	0.827
RF	0.902	0.821	0.844
L2	0.892	0.890	0.877
SVM	0.894	0.785	0.818
MDL-CPI	0.975	0.943	0.923
GNN	0.978	0.938	0.929
GCN	0.975 ± 0.004	0.921 ± 0.008	0.927 ± 0.006
GraphDTA	0.974 ± 0.004	0.927 ± 0.015	0.912 ± 0.023
TransformerCPI	0.988 ± 0.002	0.952 ± 0.006	0.953 ± 0.005
AMMVF-DTI (this work)	**0.990**	**0.962**	**0.960**

**Table 4:** The performance comparison of our model AMMVF-DTI with existing models on the DrugBank dataset. The best performance values are highlighted in bold.

Model	AUC	Precision	Recall
RWR	0.7595	0.7046	0.6511
DrugE-Rank	0.7591	0.7070	0.6289
DeepConv-DTI	0.8531	0.7891	0.7385
DeepCPI	0.7003	0.7006	0.5563
MHSADTI	0.8628	0.7706	0.7918
AMMVF-DTI (this work)	**0.9570**	**0.9034**	**0.9084**

**Table 5:** Summary of the three datasets used in this study, namely, human, *C. elegans*, and DrugBank.

	Human	*C. elegans*	DrugBank
Number of drugs	1052	1434	6707
Number of target proteins	852	2504	4794
Number of total samples	6728	7786	37,102
Number of positive interactions	3364	3893	18,398

## Data Availability

The data presented in this study are available in Supplementary Materials.

## Supplementary Materials

The Python code for our AMMVF-DTI model is available as open source and can be downloaded from the following website: https://github.com/frankchenqu/AMMVF (accessed on 9 September 2023).
